# Advanced materials for cancer treatment and beyond

**DOI:** 10.3389/fphar.2025.1557155

**Published:** 2025-03-05

**Authors:** Lei Zhang, Yanan Wang, Yangjia Li, Zhe-Sheng Chen, Chaohua Hu

**Affiliations:** ^1^ College of Life Sciences, University of Chinese Academy of Sciences, Beijing, China; ^2^ College of Life Sciences, Fujian Agriculture and Forestry University, Fuzhou, China; ^3^ State Key Laboratory of Structural Chemistry, Fujian Institute of Research on the Structure of Matter, Chinese Academy of Sciences, Fuzhou, China; ^4^ National Engineering Research Center for Sugarcane, Fujian Agriculture and Forestry University, Fuzhou, China; ^5^ Department of Pharmaceutical Sciences, College of Pharmacy and Health Sciences, St. John’s University, Queens, NY, United States

**Keywords:** reversal of multidrug resistance (MDR), tumor microenvironment (TME)-responsive material, controllable treatment of cancer, materials for enhancing immunity, integrated cancer diagnosis

## Abstract

Conservative anti-cancer treatment represented by chemotherapy and surgery lacks tumor-specificity and could hardly resolve the problems associated with multidrug resistance (MDR) in cancers. Novel therapeutic materials in cancer treatment, such as those with anti-MDR or controllable treatment features, represent a significant trend due to their advantages of high and specific efficacy and timely intervention of cancer progress. In addition to their excellent biocompatibility and specificity, they can be utilized in therapies that require ease of operation, provided they are designed with high detection sensitivity. In this review, we summarize a series of recently developed materials that exhibit these advantages, including immune-enhancing and tumor microenvironment (TME)- responsive materials, and those with integrated therapeutic and imaging capabilities. We also introduce advanced modification approaches that can impart essential targeting functionalities to these materials.

## 1 Introduction

Cancers represent a category of challenging diseases that are prone to develop MDR and recurrence, leading to low survival rates among patients (Vaidya et al., 2020). These diseases can arise from a multitude of factors and involve complex biological mechanisms. Furthermore, malignant tumors are often protected by physiological and physical barriers, such as mechanisms of drug resistance, immune tolerance, and the tumor microenvironment, which pose significant obstacles to effective treatment and potential cure.

In this context, the exploration of chemotherapeutic agents and materials-based anti-cancer drugs must consider not only comprehensive factors such as targeted drug delivery and systemic immunity but also the individual characteristics of each patient. This includes considerations of the specific cancer type, the nature of the MDR, the stage of cancer progression, and the mutation genotype associated with the tumor. Such individualized approaches necessitate rapid diagnosis and timely therapeutic interventions to enhance treatment efficacy and improve patient outcomes.

**FIGURE 1:**
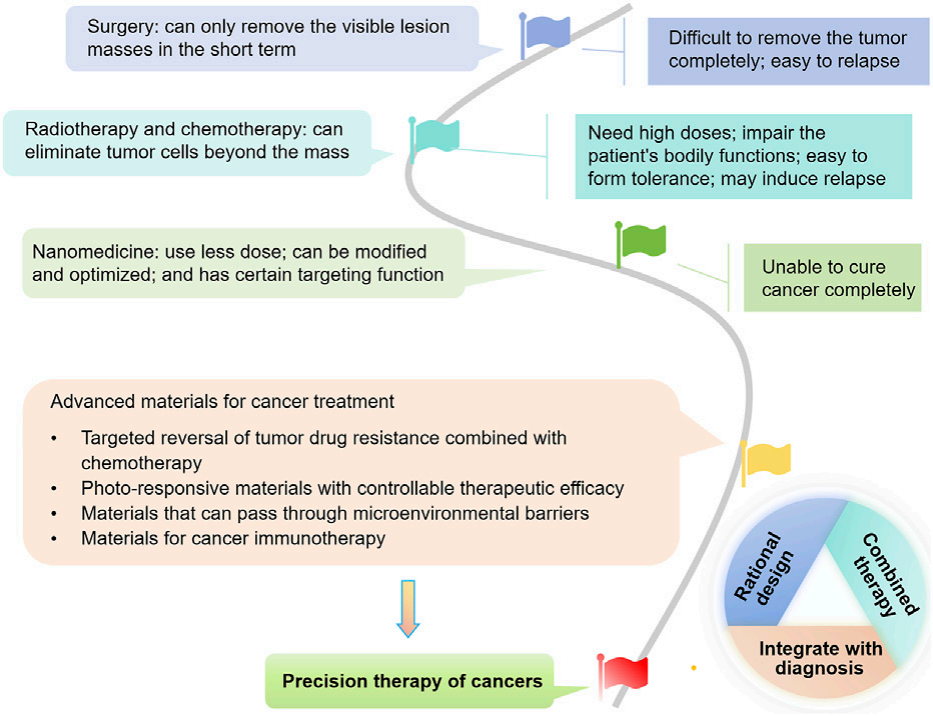
The tree map illustrating the development of strategies for cancer treatment, with a focus on advanced materials which hold promises for reversing drug resistance, enabling controllable phototherapy, overcoming tumor microenvironment (TME) barriers, and activating immunity. They are also promising for rational design, combined therapy, and integrated diagnosis and treatment, ultimately benefiting precision cancer therapy.

Currently, a variety of unique nanomaterials with distinct advantages are being explored, aimed at addressing the issues encountered with traditional nanomaterial applications (Bueschbell et al., 2022; Bukowski et al., 2020) (Figure 1). The diversity of these materials and their significant characteristics and benefits offer more possibilities for flexible combinations among materials, facilitating the creation of new nanomaterials that can achieve precise treatment (Balwe et al., 2024; Gupta and Sharma, 2024; Singh et al., 2021; Wang et al., 2023). In combined therapeutic nanomaterials that have been successfully developed, those with the activity to overcome MDR, promote generation of reactive oxygen species (ROS), benefit in-site-treatment, facilitate tumor-environment entrance or enhance immuno-therapies, have exhibited great potential for anti-cancer treatment with improve efficacy and sustainability (Gupta and Sharma, 2024; Karimi Alavijeh and Akhbari, 2024; Nakamura et al., 2022; Nejabat et al., 2024). Beyond, the materials with integrated imaging technology can bring about accurate diagnosis and with ease the practical application of the advanced materials, which largely declined time and economic cost during anticancer-therapy, meanwhile, the patient’s pain and discomfort will also be reduced (Wong et al., 2019).

The rational design of nanoparticles (NPs) with efficient therapeutic activities employs a strategic approach that integrates principles from chemistry, biology, and materials science to tailor nanomaterials with specific properties optimized for therapeutic applications (Jia et al., 2025; Zhu et al., 2025). This method focuses on refining the design of nanoparticles to enhance drug delivery, improve cellular uptake, and increase biocompatibility, ultimately resulting in more effective treatment outcomes and minimized side effects. Through this careful customization, researchers aim to create nanoparticles that are not only functional but also precisely aligned with the needs of targeted therapies.

This review highlights the potential of novel nanomaterials that not only address various challenges in traditional cancer treatments but also integrate advanced imaging technologies. These innovations facilitate more precise diagnosis and personalized therapeutic approaches, significantly enhancing treatment efficacy while reducing treatment duration and patient discomfort.

## 2 Materials with the properties to inhibit MDR cancers

**FIGURE 2:**
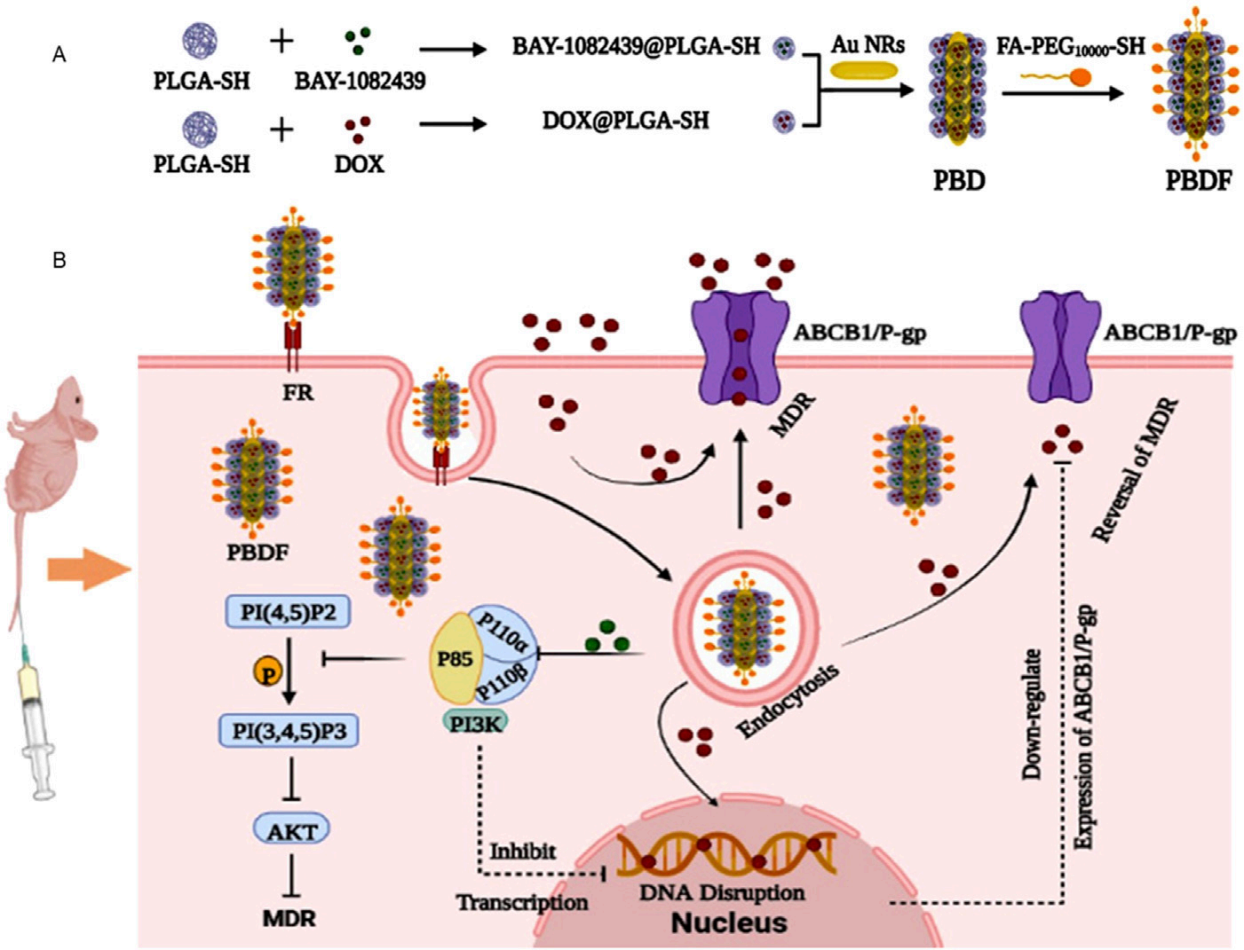
Schematic illustration of PBDF NPs with enhanced anti-cancer functions. **(A)** Structure of PBDF NPs. PLGA-SH encapsulating BAY-1082439 or DOX were prepared as BAY-1082439@PLGA-SH or DOX@PLGA-SH NPs, respectively. These NPs were then grafted onto the Au NR cores, which were modified with FA-PEG_10000_-SH, resulting in the formation of PBDF NPs. **(B)** The rationale for PBDF NPs in inhibiting cancers with PI3K promoted MDR. The KB-C2 cancer cells overexpressing ABCB1 exhibited MDR, causing the majority of DOX to be extruded from the cells. By utilizing PBDF NPs, both DOX and BAY-1082439 are specifically delivered into tumor cells through folate receptor FR mediated targeting. This process is followed by the internalization of the nanoparticles, PLGA degradation, and drug release. Subsequently, BAY-1082439 inhibits the activity of PI3K 110 subunits, P110α and P110β, which leads to the suppression of target gene transcription and downregulation of P-gp expression. As a result, the accumulation of DOX within KB-C2 cells increases, promoting its entry into the cell nuclei, thereby interfering with DNA replication and promoting apoptosis and cell death (Lin et al., 2023). PLGA: Poly (lactic-co-glycolic acid); DOX: doxorubicin; FA: folic acid (folate); FR: folate receptor; PEG: polyethylene glycol.

MDR has always been considered as a vital factor for preventing cancer treatment. At present, materials showing the activities to target MDR-associated proteins in cancers are being increasingly explored. However, due to the limitation of current knowledge of MDR mechanisms and regulation proteins, these studies are somewhat restricted. Till now, materials targeting specific MDR associated pathways or targets are not widely reported. In our laboratories, we revealed phosphatidylinositol 3-kinase (PI3K) 110 alpha and beta as new MDR related enzyme units, which upregulate expression of ATP-binding cassette proteins ABCB1 (also known as P-glycoprotein P-gp) and ABCG2 in cancer cells (Zhang et al., 2020). And on this base, we developed combined and tumor targeted nano-materials (namely PBDF) with enhanced activity to inhibit cancer cells with MDR mediated by PI3K 110 alpha and beta that promoting ABCB1 expression (Figure 2) (Lin et al., 2023).

Till now, some materials that can change the expression level of tumor-associated proteins were revealed to have the ability to inhibit MDR cancers. Based on symmetrical Se-esters showing potent anticancer activity, combined application with doxorubicin resulted in affection on the ATPase activity of ABCB1 (P-gp) (Racz et al., 2023). With the modulation functions on specific tumor-associated factors, CS-PLGA-PHL was able to downregulate the expression of matrix metalloproteinases MMP-2 and MMP-9 genes and proteins significantly in drug-resistant cell lines (Yuan et al., 2024). Nitazoxanide as a potent adjuvant in colorectal cancer (CRC) chemotherapy, could reverse MDR in CRC cells by downregulation of Wingless/Int-1 (Wnt)/beta-catenin signaling pathway (Hemmati-Dinarvand et al., 2022); therefore, it could be considered as decent partner in developing materials for treatment of MDR cancers. However, more specific mechanisms and regulation targets remain to be clarified in respect of developing the materials working on MDR cancers and tumor-targeting approaches are yet to be optimized.

## 3 Photo-responsive nano-materials with controllable therapeutic efficacy

Photo-responsive nano-systems such as photothermal, photodynamic or photocatalytic materials can efficiently convert energy from light irradiation into heat, light emission at specific wavelengths, and generation of ROS through mechanisms such as electronic transitions, energy resonance, or generation of high-energy electrons and holes (Agostinis et al., 2011; Ethirajan et al., 2011). Compared with non-photo-responsive materials, these materials can be developed with high efficacy to serve in site and controllable treatment of tumors, when certain wavelength of light was provided. Due to the localized hyperthermia, ROS generation within the tumor cells, the necrosed tumor cells can also release tumor associated antigens, which could enhance the tumor-response of the immune system. The most remarkable characteristic of photothermal therapy (PTT) or photodynamic therapy (PDT) lies in their ease of modulation and minimal invasion during tumor treatment. Not only the wavelength and intensity of the light resource, but also the delivery and release format of the materials can all be well designed and controlled to suit different requirements of therapies. Besides, the temperature varies of the tumors from the surrounding normal tissues, or the fluorescence/phosphorescence generated during PTT or PDT, can be applied to optimize real-time imaging tracking of the material accumulation within the tumors, largely reducing the time costs for accurate treatment of cancers.

The materials used for PTT include various photothermal responsive substances such as gold, palladium (Pd), platinum (Pt), and carbon NPs. Gold NPs are particularly effective due to their ability to absorb near-infrared (NIR) light and convert it into localized heat, making them ideal for targeted cancer treatments while sparing healthy tissue (He et al., 2022). Similarly, Pd and Pt NPs serve as catalysts that enhance the photothermal response (Cen et al., 2022). Carbon NPs, including carbon quantum dots (CQD) and graphene showing decent biocompatibility, improve heat distribution due to their excellent thermal conductivity (Yuan et al., 2023). By combining these materials in PTT, researchers aim to enhance precision treatment and efficacy in cancer therapy and other biomedical applications.

PDT employs various photosensitizing materials, such as 5-aminolevulinic acid, porphyrin-based compounds, and nanomaterials like gold NPs and carbon nanomaterials (Ghoochani et al., 2023; Pustimbara et al., 2024). In tumor treatment, these materials rely on their ability to generate ROS under specific wavelengths of light, which can selectively damage cancer cells. Photosensitizers (PSs) are typically designed with small sizes and can be internalized by tumor cells and, when activated, they release energy that induces cancer cell apoptosis or necrosis. Moreover, the incorporation of nanomaterials with functional ligands or bio-utilizable molecules can improve their tumor targeting drug delivery efficiency and uptake efficiency, enhance their light absorption, and facilitate tumor-targeting treatment, effectively minimizing damage to healthy tissues (Anitha et al., 2024; Wang D. N. et al., 2024).

Integrated therapy based on PTT or PDT were developed rapidly, which large benefit anti-cancer therapy. Recently, a novel “three-in-one” multimodal nanocatalytic therapy using a multifaceted 2D black phosphorus/Pd nanosheet platform was developed for enhanced tumor-specific treatment through synergistic photothermal, photodynamic, and chemodynamic mechanisms while minimizing harm to normal tissues (Fu et al., 2022). In another case, a novel PdH@MnO_2_/Ce6@HA yolk-shell nanoplatform is developed to enhance hydrogen therapy and PDT in cancer treatment by facilitating stable hydrogen storage, efficient oxygen generation, and targeted delivery to cluster of differentiation 44 (CD44)-overexpressing melanoma cells (Wang et al., 2022). Ghoochani et al. demonstrates that zinc porphyrin encapsulated in MIL-101 (Zn [TPP]@MIL-101) effectively eradicates MCF-7 breast cancer cells through PDT, achieving half maximal inhibitory concentration (IC_50_) values of 14.3 mg/mL under light and 81.6 mg/mL in the dark (Ghoochani et al., 2023). Current integrated therapies based on PTT or PDT face limitations such as insufficient tissue penetration and potential damage to surrounding healthy tissues. Additionally, their effectiveness can be hindered by the development of resistance in targeted cells and the variability in light activation conditions.

Both PDT and PTT are effective phototherapeutic methods for eliminating tumor cells. However, controlling the temperature of non-tumor cells and reducing cytotoxicity of PTT have become important issues during material design and application. PDT efficacy is often limited by insufficient oxygen concentration in the environment, while the temperature elevation caused by PTT can accelerate local blood circulation and alleviate hypoxia to some extent (Han et al., 2016; Li et al., 2019; Xiao et al., 2013); therefore, combined PTT-PDT might be applicable for efficient anti-cancer therapy. Besides, PDT has the side-effect of promoting metastasis due to ROS production, which should be considered for optimization before clinical application.

## 4 Novel designed materials facilitating entrance of TME

The TME plays a crucial role in tumor progression by providing a supportive environment for cancer cell growth and facilitating communication between cancer cells and surrounding stromal cells. It also contributes to immune evasion, creating challenges for effective treatment strategies (Shukla et al., 2024). In anti-cancer therapy, the TME can significantly influence the efficacy of cancer drugs by affecting their distribution, metabolism, and the therapeutic response of cancer cells.

On one hand, TME are deemed as a natural protective screen against therapeutic agents. On the other hand, researchers are carrying out tempts for construct therapeutic drugs/materials that can regulate, target or even response to TME (Zhou et al., 2024), which may be more tumor-specific and largely reduce the side-effect on normal tissues. Till now, development of TME-responsive materials is somewhat limited but keeps in proceeding. For example, hydrogen peroxide (H_2_O_2_) has high concentration in TME, bringing about the developments of H_2_O_2_-responsive materials and their potential in overcoming TME-induced hypoxia (Yang et al., 2020). The extracellular vesicles (EVs) play important roles during the information exchanges between cancer cells and surrounding microenvironment. EV-ncRNAs have been considered as potential biomarkers and therapeutic targets for developing novel materials for cancer therapy (Zhou et al., 2024). By modeling TEM environment featuring chemical and mechanical gradients, interfaces and fluid flow, materials could be designed and applied to target, dissect and rebuild TME, facilitating the exploration of precision medicine (Wolf et al., 2019).

## 5 Combined materials for enhanced immunotherapy

MDR in cancers calls for effective immunotherapeutic strategies urgently to achieve effective and long-lasting protection against cancers. The major hinders of immunotherapy for clinical applications are its low response rate, insufficient therapeutic potency, adverse side effects and nonspecific delivery (Yang et al., 2024). Nanomaterials, which can enhance immune response, have shown their advantages in immune-activation and anti-cancer combat.

Recently, Wu et al. designed Au-DP@FeCaC NPs which could reprogram tumor associated macrophages (TAMs) from immunosuppressive M2 to immunostimulatory M1, inhibit P-gp expression, and realize the immunotherapy-mediated highly efficient chemotherapy of MDR cancer (Wu et al., 2022). Li et al. reported immunomodulator comprising an IR-1061 dye-encoded NIR-II porous cubic AuAg nanoshell (pcNS) loading a Toll-like receptor 7 agonist (R837) in phase change materials, subsequently modified with hyaluronic acid (HA). The resultant RP@IR-pcNS@HA NPs controllably delivered and released R837 to tumor sites in response to NIR-II photoirradiation, and perform plasmonic hyperthermia therapy for direct ablation of primary tumors while eliciting robust anticancer immune responses (Li et al., 2024). Based on a series of clinical data, Mo et al. claimed neoadjuvant therapy using immune checkpoint inhibitors plus chemotherapy is a promising novel approach, which may promote studies of combined materials for immunotherapy against cancers (Mo et al., 2024). Through precise biochemical reactions, novel catalytic materials can modulate the immunosuppressive TME and show great potential in augmenting catalytic immunotherapy. Advanced materials can be designable and functionalized in every potential aspect to active and modulate immune system, showing great prosperity in combined immunotherapy in future.

**FIGURE 3:**
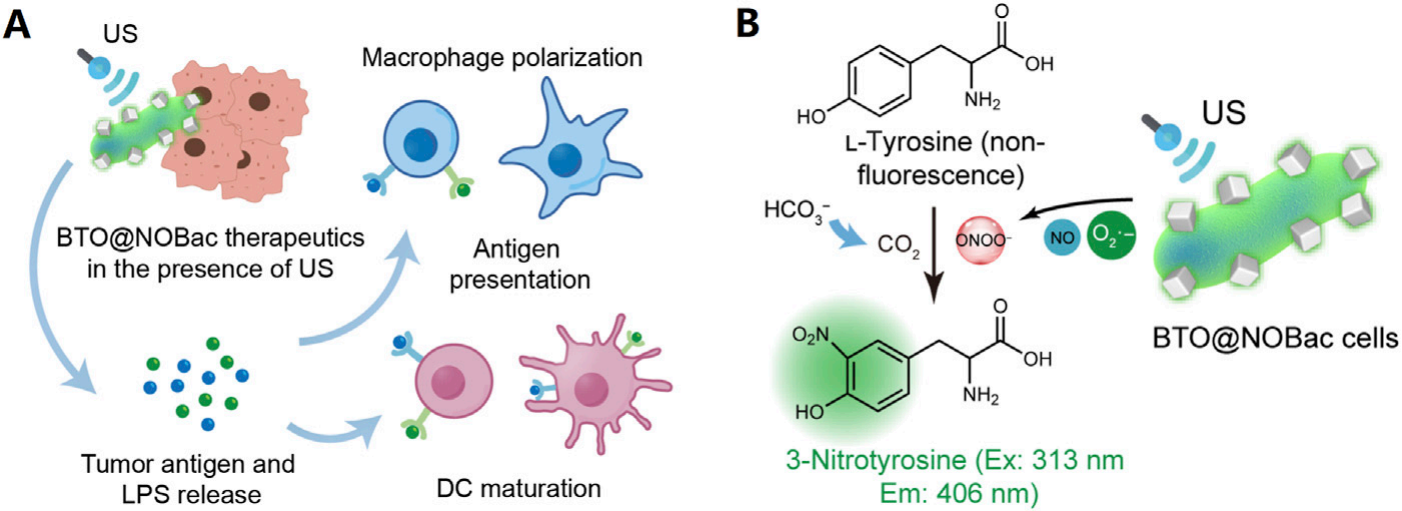
BTO@NOBac for tumor piezocatalytic therapy with sustained antitumoral immunity. **(A)** Mechanism schematics of dendritic cell (DC) maturation and macrophage polarization, induced by the sonopiezo-catalytic therapeutic of BTO@NOBac. Under ultrasound irradiation, superoxide anions produced by the piezocatalytic reaction of BTO NPs can immediately react with nitric oxide (NO) generated from NOBac, leading to the formation of highly oxidative ONOO^−^ species in a cascade reaction. This process results in significant tumor piezocatalytic therapeutic efficacy and induces prominent and sustained antitumoral immunoactivation simultaneously. **(B)** Mechanism of the method for the detection of ONOO^−^ at solution level. The generation of ONOO^−^ by BTO@NOBac under ultrasound irradiation can be evaluated by using L-tyrosine. In the presence of carbon dioxide, ONOO^−^ nitrates L-tyrosine to form 3-nitrotyrosine, which emits characteristic fluorescence with an excitation wavelength of 313 nm and an emission wavelength of 406 nm (Wang L. P. et al., 2024).

Living therapeutics involves using engineered microorganisms, such as NOS-engineered *Escherichia coli* (NOBac), hybridized with sonopiezocatalytic BaTiO_3_ NPs (BTO NPs) to promote tumor-targeted accumulation and antitumor therapy (Figure 3). When activated by ultrasound, the piezocatalytic reaction of BTO NPs created superoxide anions which immediately react with nitric oxide (NO, generated from NOBac) to produce highly oxidative peroxynitrite ONOO^−^ species in cascade, resulting in robust tumor piezocatalytic therapeutic efficacy, realizing sustained antitumoral immunoactivation (Wang L. P. et al., 2024).

## 6 Modification of tumor-targeting materials for accurate therapies

Upon entering the body, both traditional chemotherapy agents and NPs encounter natural physiological barriers, with the blood-brain barrier and the peritoneal membrane being primary obstacles (Sharma et al., 2022). Although nanomaterials can accumulate in tumor tissues due to the enhanced permeability and retention (EPR) effect, they also show accumulation in non-target tissues (Bjornmalm et al., 2017; Sharma et al., 2022). The interstitial fluid and the TME pose significant hurdles for NPs (Daly et al., 2020). Due to the interstitial fluid pressure, access of NP to tumor cell was limited, while convective interstitial flow can accelerate the clearance of NPs from the tumor cell surface (Abyaneh et al., 2020). Additionally, the interstitial fluid comprising mononuclear phagocytic system (MPS) that can remove NPs from the cell surface (Sharma et al., 2022). Selectivity of cell membranes can further hinder the entrance of NPs into intracellular compartments (Kodiha et al., 2015).

Materials with precise targeting properties can be designed to target biomarkers in solid tumor tissues or cycling tumor cells (CTCs). Tumor related proteins, vascular tissues, and TME can all serve as targets for developing tumor-targeting materials. New materials have remarkable characteristics in designability, along with improvements in size (to suit *in vivo* cycling, cell uptake, nuclear entrance, etc.), surface charge (to increase affinity with tumor cell membrane), targeting-ligand modification (to enhance tumor specificity), environment (pH, temperature, etc.) responsive activity, bioavailability (by introducing biomaterials, biomolecules, etc.), and other aspects such as recognition and specifically inhibit checkpoint to enhance immunotherapy, which have demonstrated the key roles of novel materials as necessary components for targeted anticancer drugs and the drug delivery vehicles (Feng et al., 2024). Most materials were designed with combined factors to achieve the best targeted anti-cancer therapeutic efficiency.

Some materials like hydrogel were designed as carriers to enhance the drug permeability. They can be modified with suitable sizes, proper hydrophilic-hydrophobic properties, targeting functions, and phase-transition ability in response to environment changes. The swelling behavior provides them with efficient drug-loading capability and controllable drug-release behavior (Guo et al., 2025). Li et al. developed an oral hydrogel (OPBP-1) with intestinal drug permeation using N, N, N-trimethyl chitosan (TMC) NPs (Cheng et al., 2018; Li et al., 2021).

In therapeutic strategies utilizing the EPR effect, particles smaller than 300 nm exhibit effective tumor retention (Subhan et al., 2021). Surface modifications can be employed, such as linking with monoclonal antibodies and aptamers to reduce the clearance of NPs *in vivo*, as well as to decrease toxicity to normal organs (Poon et al., 2019). After the modification of liposomal NPs with cleavable radionuclide anchors, their accumulation in the spleen and other organs was effectively reduced (Llop and Lammers, 2021). Red blood cell (RBC) membranes can be incorporated into NPs to form RBC-NP complexes that aid in tissue penetration (Glassman et al., 2020). These complexes exhibit reduced accumulation in the liver and spleen (Zhang, 2016). In addition to optimizing the intrinsic properties of the materials, auxiliary strategies can also enhance efficacy. For instance, Xiong et al. reported that localized ultrasound exposure can increase the extravasation of nanomaterials (Xiong et al., 2017).

**FIGURE 4:**
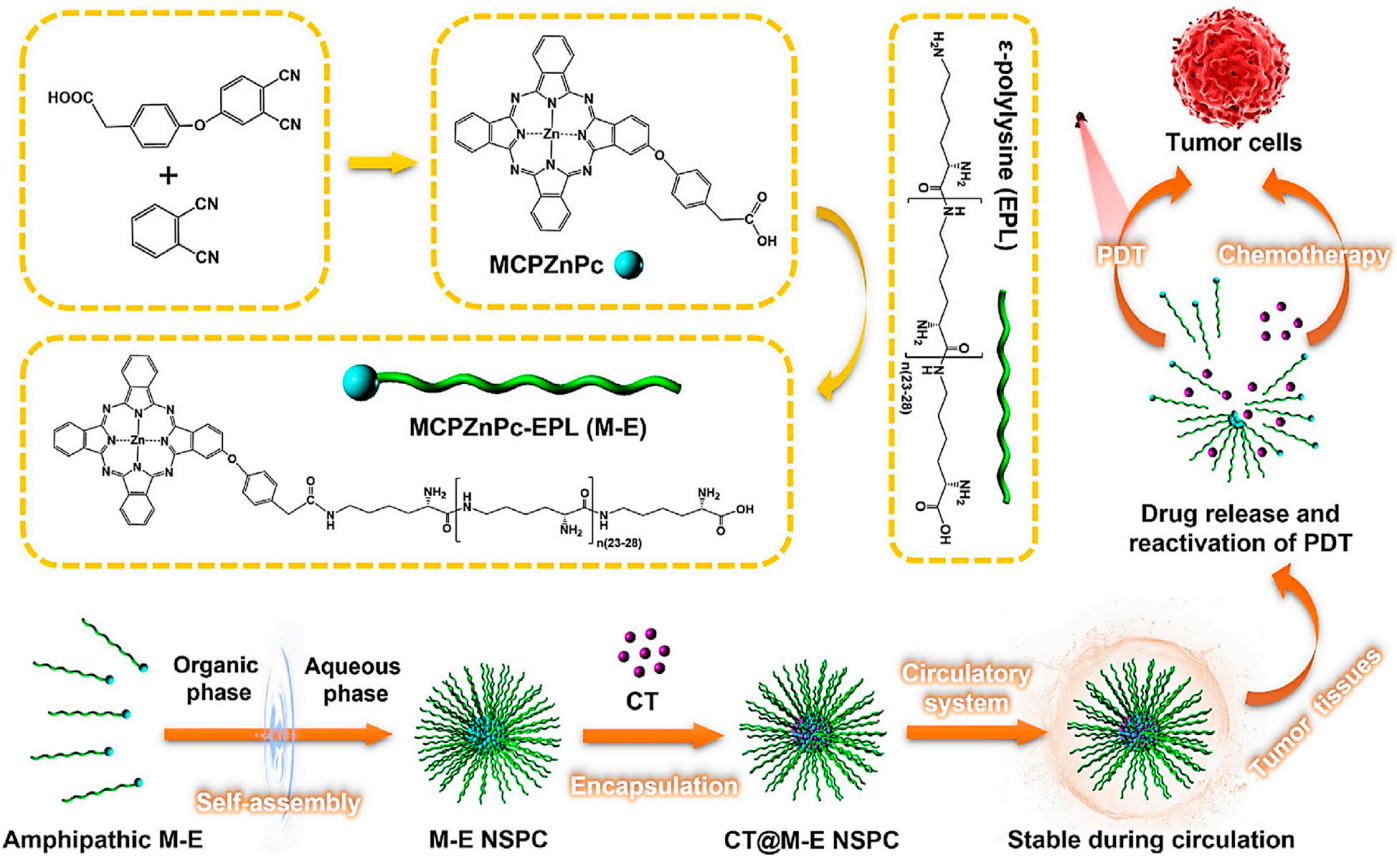
Development of CT@M−E NSPC for combined PDT-chemotherapy against cancers. CT@M−E NSPC have high stability against serum disintegration and showed enhanced PDT and chemotherapeutic efficacy for cancer treatment (Chen J. et al., 2023).

Serum components frequently disintegrated the composites of nanomaterials, which can result in off-target effects. Our research group developed a nanostructured self-assembling peptide-photodynamic agent carrier (NSPC) comprising conjugated ε-poly-L-lysine and phthalocyanine derivatives (MCPZnPc) for the delivery of chemotherapeutics (CT) (Figure 4). The resultant particle, CT@ME NSPC was highly stable in serum, which effectively protected the chemotherapeutic agent while facilitating a synergistic PDT and chemotherapeutic therapeutic efficiency (Figure 3) (Chen J. et al., 2023).

The accumulation of NPs (such as liposomes) in tumor tissues can be effectively increased by adding hydrophilic coating materials, such as polyethylene glycol (PEG) to their surfaces (Khan et al., 2020; Rommasi and Esfandiari, 2021). Another strategy involves enabling NPs to degrade the extracellular matrix, for instance, the incorporation of pH-triggered, degradable collagenase can enhance the permeability and cytotoxicity of the nanomaterials (Wang et al., 2018; Xu et al., 2020). Encapsulating NPs can help mitigate the corona effect and reduce immune clearance, potentially aiding in the subcellular localization of the nanomaterials (Bevilacqua et al., 2021).

In addition to biomarkers such as vascular endothelial growth factor receptor 2 (VEGFR2), arginine-glycine-aspartic acid (RGD), folate receptors, and other tumor-associated proteins, a novel biomimetic approach mimics the properties of human viruses. This strategy enhances the targeting efficiency of materials, helps them evade host immune clearance, and promotes cellular uptake. Wu et al. mimicked the structure of the rod-shaped tobacco mosaic virus (TMV), which can disrupt the endoplasmic reticulum, by designing small and repetitive subunit capsid-subunit-mimetic dendrons (CSMDs) (Wu et al., 2020). Our research group employed non-pathogenic *E. coli* to target tumor delivery of PSs, enhancing PDT for tumors (Dai et al., 2020).

Small molecules like miRNA, which are specifically overexpressed in various cancer cells, are also utilized as tumor biomarkers. For example, miRNA-21 has a good application prospect for proliferation inhibition and prognosis judgment of breast cancer (Liang et al., 2022; Yurdabak Karaca et al., 2021). Karaca et al. reported the preparation and characterization of catalytic gold/poly (3,4-ethylenedioxythiophene)/platinum (Au/PEDOT/Pt) micromotors, demonstrating their application in detecting the cancer biomarker miRNA-21 and their potential for cancer treatment. The use of anti-miRNA probe-immobilized micromotors showed high selectivity for cancer cells (specifically MCF-7 and SJSA-1) (Yurdabak Karaca et al., 2021).

## 7 Rational design of materials with dual therapeutic and diagnostic properties

The rational design of materials with dual therapeutic and diagnostic properties—often referred to as theranostics—emerges as a transformative advancement in the field. Theranostic materials are engineered to simultaneously deliver therapeutic agents and allow for real-time monitoring of their effectiveness through diagnostic imaging (Ren et al., 2015; Sharma et al., 2022). By integrating both therapeutic and diagnostic functionalities, these innovative materials offer the potential for personalized medicine, where treatment can be tailored based on immediate feedback from diagnostic assessments, thus enhancing overall patient care and treatment precision. Together, these approaches exemplify the significant promise of nanotechnology in revolutionizing medical therapies and diagnostics.

Materials with integrative therapeutic and diagnostic properties guarantee early diagnosis and treatment of cancers, and have wide application necessity for “real-time” imaging during surgery. Theranostic materials also show great potential in managing precancerous conditions like chronic inflammation, Crohn’s disease, ulcerative colitis, and colorectal polyps, which play crucial roles in cancer development and progression (Coussens and Werb, 2002; Nigam et al., 2023). These innovative materials facilitate early detection through enhanced imaging capabilities and enable targeted therapy that minimizes side effects while improving treatment efficacy. By combining both functions, they allow for timely monitoring of disease progression and localized treatments, ultimately reducing the risk of progression to cancer. Continued collaboration among researchers is essential to advance these materials from the lab to clinical use, enhancing patient care and outcomes in oncology. As mentioned, PDT and PTT materials are well known selections for combining tumor treatment and detection. Other technologies are associated with energy transition, fluorescence-enhancing strategies for varied requirements such as treatments of deep tumors with enhanced ROS production and imaging efficiencies.

In addition to these approaches, materials triggered by pH, hypoxia, and elevated levels of glutathione in TME have great potential in accurate diagnosis and anticancer treatment (Chu and Stochaj, 2020; Meng et al., 2017; Sharma et al., 2022). These factors can not only facilitate tumor detection but also trigger the release of drugs encapsulated in NPs (Gao et al., 2021; Zhang et al., 2021). The pH level of the TME is typically lower, ranging from 6.5 to 6.8, compared to normal tissue, thus becoming a triggering condition for the functionality of numerous materials *in vivo* (Li et al., 2018). Base on PS hypericin (Hyp), Zhao et al. developed a convertible UCNP@MnOx-Hyp system that reacts to intracellular glutathione and H_2_O_2_, leading to the reduction of materials and the release of Mn^2+^ and Hyp within tumor cells; the PDT effectiveness of this system was enhanced by *in situ* crosslinking (Zhao et al., 2023). Based on the material’s sensitivity to glutathione (GSH) present in the TME, Liu et al. designed multi-shell NPs exhibiting strong PTT effects (Liu et al., 2021).

Precisely targeting cancer stem cells (CSCs) can enhance the overall effectiveness of cancer therapies. Materials with similar functions are mainly carbon-based, such as metal fullerenes and graphene oxides. For example, Yu et al. designed a polyethylene glycol carbon nanohorn (PCNH) that can induce CSCs to differentiate into cells more easily cleared through elevating intracellular ROS levels (Yu et al., 2020).

Toward the tumor associated protein such as programmed death-ligand 1 (PD-L1) playing a critical role in immune checkpoint, materials modified with antibodies or aptamers targeting these proteins can be designed to achieve specific recognition, enhanced chemo- or immune-therapy and signal feedback (Ren et al., 2015; Sharma et al., 2022).

By targeting biomembrane systems like mitochondria and the endoplasmic reticulum (Otvagin et al., 2022), and organelles like lysosomes (Choi et al., 2017; Han and Choi, 2021), materials can be designed with enhanced efficiency to inhibit tumor cells. For instance, the nanomaterial TPP-UCNPs@MOF-Pt designed by Chen et al. can localize at the mitochondria and, upon excitation with near-infrared light, causes severe depolarization of the mitochondrial membrane while activating apoptotic pathways, thereby amplifying the therapeutic effect (Chen Y. et al., 2023). Zhang et al. designed autophagy-activating fluorescent PSs that incorporate two types of dual-emission aggregation-induced emitters (AIEgens—TPAQ and TPAP). These PSs can generate ROS upon autophagy activation to counteract the protective effects of autophagy in cancer starvation therapy (Zhang et al., 2023). The lysosomal environment with a pH of approximately 5 is also conducive for activating pH-responsive materials (Zhang et al., 2021). Cheng et al. designed a dual-targeting strategy for nanomaterials that enhanced cell membrane permeability by targeting membrane and mitochondria through a hydrophilic PEG linker connected with the PS protoporphyrin IX (PpIX), resulting in a significant increase in PDT efficacy (Cheng et al., 2019).

Furthermore, customizing treatment plans based on individual physiological conditions (genetic information, age, gender, body weight and composition, body temperature, blood glucose level, blood pressure, metabolic rate, hormonal levels, immune function, organ function, and etc.) is another approach to improving precision medicine. For instance, Liu et al. developed an optimized irradiation scheme for PTT based on individual differences, effectively highlighting the advantages of customized PTT while maintaining high therapeutic efficacy and survivability (Liu et al., 2021).

It is known that most anti-tumor drugs lack stability in *in vivo* environment. To resolve this problem, a variety of materials with enhanced ability to protect molecules like PSs have been developed. For instance, the nano-to-micro sized hydrogel loading systems, which are ideal for rational design and modifications, can be endowed with decent permeability and target capability (Mastropietro et al., 2012). More importantly, the drug-loading system based on hydrogel materials can be well protected against various enzymes and factors, to avoid degradation and achieve prolonged accumulation within tumor cells.

At present, we are seeking new materials based on nano-to-micrometer sized biochar for anticancer drug development. Biochar is highly stable and biocompatible because it can be prepared from plant stems and leaves (Abdullah et al., 2023). It has the potential of being used as vectors for drugs or NPs, to enhance the stability and applicability of the passengers in anti-cancer therapy.

**FIGURE 5:**
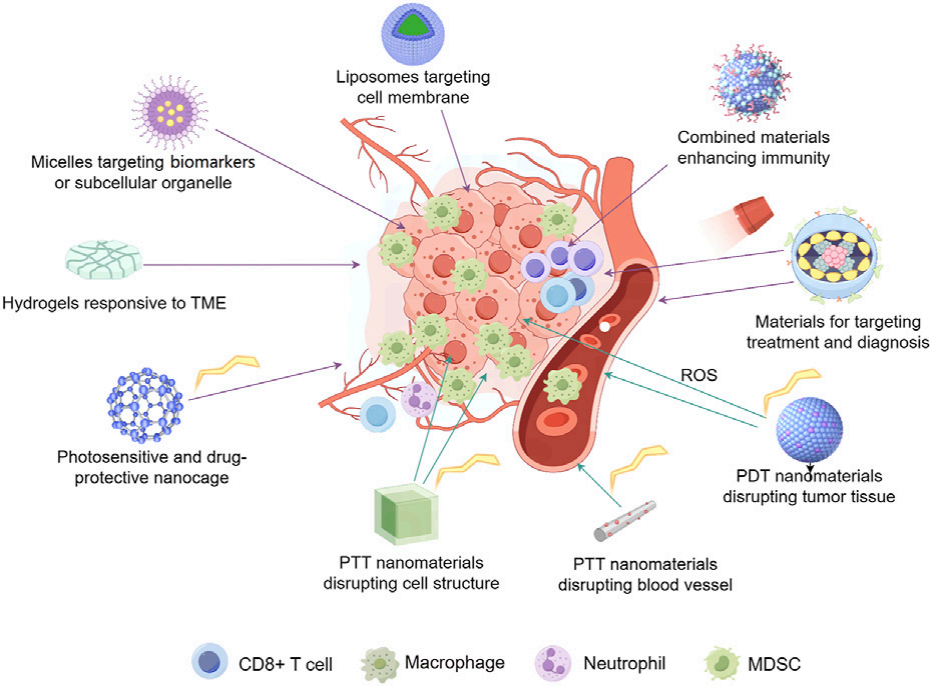
Rationale design of nanomaterials for anti-cancer therapy. These materials are normally developed with targeting and environment-responsive properties. Morphological characteristics are critical for suitability in permeation and drug-release. Factors like immune system, blood vessels in tumor tissues, TME, cell skeleton and subcellular organelle, as well as biomarkers distributed in different positions of cancer cells, pH, and etc. are comprehensively assessed for target therapy on a specific patient. Real-time imaging techniques were combined for precise diagnosis.

Nanomaterials are increasingly employed in the diagnosis and treatment of tumors due to their superior biological penetration capabilities. Due to their flexibility in design for the use of different needs including drug release and response to environment, high sensitivity in target detection, effective therapeutic performance, they are now used for various purposes (Table 1). However, there still exist disadvantages such as biological toxicity, over-short accumulation in tumors or over-long accumulation in bodies, or genitive impact on immunity (Ryman-Rasmussen et al., 2006). For example, metallic NPs have been shown to induce the production of cytotoxic factors, provoke inflammatory responses, and generate ROS, which may be detrimental to the biomolecules and organisms in non-cancer cells (Teleanu et al., 2018; Valdiglesias et al., 2016). As the functions associated with disease diagnosis and treatment are kept integrated into current nanomaterials, it is crucial for researchers to focus not only on the specificity and functionality of new materials but also to carefully consider the underexplored toxicological properties of various materials, along with the implications of their residuals and metabolic pathways once they enter the body. The toxicity of NPs is often influenced by factors such as element type, chemical activity, size and shape, surface charge, and other characteristics like clearance period and so on. Furthermore, biomarkers and their related expressions can vary at different stages and degrees of tumor progression, making precise identification and comprehensive treatment particularly challenging under these conditions (Sharma et al., 2022). During the exploration of therapeutic materials, particularly those for clinical use, these factors should at least be considered (Figure 5).

**TABLE 1:** Representative categories of advanced materials for anticancer applications.

Category	Function	Citation	Description
Overcoming MDR	Materials with the properties to inhibit MDR cancers	Zhang et al., 2020; Lin et al., 2023	Identified PI3K 110α/β as novel MDR-related enzyme units and developed PBDF nanoparticles to inhibit PI3K-mediated MDR in cancer cells. PBDF nanoparticles synergistically target PI3K 110α/β to suppress ABCB1 expression and enhance anti-MDR efficacy
		Racz et al. (2023)	Symmetrical Se-esters combined with doxorubicin inhibit ABCB1/P-gp ATPase activity in MDR cancer cells
		Yuan et al. (2024)	CS-PLGA-PHL downregulates MMP-2/MMP-9 expression in drug-resistant cell lines
Photo-Responsive Nanomaterials	Photo-responsive materials with controllable therapeutic efficacy	Agostinis et al. (2011)	PDT using photosensitizers generates ROS under specific light wavelengths to selectively destroy cancer cells
		Ethirajan et al. (2011)	Porphyrin-based photosensitizers enable ROS-mediated apoptosis in tumors
		He et al. (2022)	Gold NPs convert NIR light into localized heat for targeted PTT.
		Cen et al. (2022)	Pd and Pt NPs act as catalysts to amplify photothermal effects
		Yuan et al. (2023)	Carbon nanoparticles (e.g., quantum dots, graphene) improve heat distribution in PTT due to superior thermal conductivity
		Ghoochani et al. (2023)	Zn-porphyrin@MIL-101 demonstrates potent PDT activity against MCF-7 breast cancer cells (IC_50_ = 14.3 mg/mL under light)
TME-Responsive Materials	Novel designed materials facilitating entrance of tumor microenvironment (TME)	Yang et al. (2020)	H_2_O_2_-responsive materials counteract hypoxia in TME for improved drug delivery
		Zhou et al. (2024)	EV-ncRNAs serve as biomarkers and therapeutic targets for TME modulation
Immunotherapy-Enhancing Materials	Combined materials for enhanced immunotherapy	Wu et al. (2022)	Au-DP@FeCaC NPs reprogram TAMs from M2 to M1 phenotype and inhibit P-gp expression
		Li et al. (2024)	RP@IR-pcNS@HA NPs release R837 under NIR-II light, inducing antitumor immunity
		Mo et al. (2024)	Neoadjuvant immune checkpoint inhibitors combined with chemotherapy show promise for immune activation
		Wang et al. (2024a)	Engineered NOBac bacteria hybridized with BaTiO_3_ nanoparticles generate ONOO⁻ via ultrasound-activated piezocatalysis for sustained antitumor immunity
Targeted Delivery Systems	Modification of tumor-targeting materials for accurate therapies	Khan et al. (2020)	PEGylation of nanoparticles improves tumor accumulation via the EPR effect while reducing off-target toxicity
		Wang et al. (2018)	pH-triggered collagenase-modified hydrogels enhance tumor penetration and cytotoxicity
		Xu et al. (2020)	Collagenase-functionalized nanoscavengers increase drug penetration and retention in deep tumor tissues
Multifunctional Theranostic Materials	Rational design of materials with dual therapeutic and diagnostic properties	Zhao et al. (2023)	UCNP@MnOx-Hyp nanoparticles enable GSH/H_2_O_2_-responsive ROS generation and real-time imaging for enhanced PDT

## 8 Concluding remarks

In conclusion, this manuscript provides an overview of recent advancements in innovative materials for cancer treatment, focusing on overcoming significant challenges such as MDR, the complexities of TME, and immune checkpoints that hinder anticancer immunoactivity. The review highlights key innovations, including photo-responsive nanomaterials with controllable therapeutic efficacy, combined materials that enhance immunotherapy, and dual-function materials that integrate therapeutic and diagnostic capabilities. These advanced materials not only promise to improve the effectiveness of existing cancer therapies but also aim to make immunotherapy strategies more efficient by enhancing targeting and reducing side effects. Looking ahead, future research should focus on optimizing these materials for clinical applications, exploring their full potential in personalized medicine, and further investigating their interactions within the TME to improve treatment outcomes. The development of new nanomaterials is crucial for enabling precise targeting and timely therapy, which can help hinder tumor progression and minimize the occurrence of drug resistance. Proper modifications to these materials may alleviate patient suffering, reduce the required dosage during administration, and decrease potential side effects. Additionally, these innovations facilitate timely assessments of treatment efficacy and prognosis, including the detection of metastasis, allowing for prompt adjustments in therapeutic strategies. By continuing to advance material science in oncology, we can enhance the precision and effectiveness of cancer therapies, ultimately leading to better outcomes for patients.

## References

[B1] Abdullah N. Mohd Taib R. Mohamad Aziz N. S. Omar M. R. Md Disa N. (2023). Banana pseudo-stem biochar derived from slow and fast pyrolysis process. Heliyon 9, e12940. 10.1016/j.heliyon.2023.e12940 36704268 PMC9871232

[B2] Abyaneh H. S. Regenold M. McKee T. D. Allen C. Gauthier M. A. (2020). Towards extracellular matrix normalization for improved treatment of solid tumors. Theranostics 10, 1960–1980. 10.7150/thno.39995 32042347 PMC6993244

[B3] Agostinis P. Berg K. Cengel K. A. Foster T. H. Girotti A. W. Gollnick S. O. (2011). Photodynamic therapy of cancer: an update. CA Cancer J. Clin. 61, 250–281. 10.3322/caac.20114 21617154 PMC3209659

[B4] Anitha K. Chenchula S. Surendran V. Shvetank B. Ravula P. Milan R. (2024). Advancing cancer theranostics through biomimetics: a comprehensive review. Heliyon 30, e27692. 10.1016/j.heliyon.2024.e27692 PMC10944277 38496894

[B5] Balwe S. G. Moon D. Hong M. Song J. M. (2024). Manganese oxide nanomaterials: bridging synthesis and therapeutic innovations for cancer treatment. Nano Converg. 11, 48. 10.1186/s40580-024-00456-z 39604693 PMC11602914

[B6] Bevilacqua P. Nuzzo S. Torino E. Condorelli G. Salvatore M. Grimaldi A. M. (2021). Antifouling strategies of nanoparticles for diagnostic and therapeutic application: a systematic review of the literature. Nanomaterials 11, 780. 10.3390/nano11030780 33803884 PMC8003124

[B7] Bjornmalm M. Thurecht K. J. Michael M. Scott A. M. Caruso F. (2017). Bridging Bio-ano science and cancer nanomedicine. ACS Nano. Oct. 24 (11), 9594–9613. 10.1021/acsnano.7b04855 28926225

[B8] Bueschbell B. Caniceiro A. B. Suzano P. M. S. Machuqueiro M. Rosário-Ferreira N. Moreira I. S. (2022). Network biology and artificial intelligence drive the understanding of the multidrug resistance phenotype in cancer. Drug resist. updat., 60. 10.1016/j.drup.2022.100811 35121338

[B9] Bukowski K. Kciuk M. Kontek R. (2020). Mechanisms of multidrug resistance in cancer chemotherapy. Int. J. Mol. Sci. 21, 3233. 10.3390/ijms21093233 32370233 PMC7247559

[B10] Cen J. Q. Huang Y. Q. Liu J. Liu Y. A. (2022). Thermo-responsive palladium-ruthenium nanozyme synergistic photodynamic therapy for metastatic breast cancer management. J. Mater Chem. B 10 (10), 10027–10041. 10.1039/d2tb01481e 36458841

[B11] Chen J. Zhou Y. Song M. Chen Y. Wang D. Huang Y. (2023b). A Serum-Stable supramolecular drug carrier for chemotherapeutics fabricated by a Peptide-Photosensitizer conjugate. J. Colloid Interface Sci. 646, 959–969. 10.1016/j.jcis.2023.05.131 37235941

[B12] Chen Y. Yang Y. Du S. Ren J. Jiang H. Zhang L. (2023a). Mitochondria-targeting upconversion nanoparticles@MOF for multiple-enhanced photodynamic therapy in hypoxic tumor. ACS Appl. Mater Interfaces 15 (15), 35884–35894. 10.1021/acsami.3c05447 37487181

[B13] Cheng H. Zheng R. R. Fan G. L. Fan J. H. Zhao L. P. Jiang X. Y. (2019). Mitochondria and plasma membrane dual-targeted chimeric peptide for single-agent synergistic photodynamic therapy. Biomater. Jan. 188, 1–11. 10.1016/j.biomaterials.2018.10.005 30312907

[B14] Cheng K. Ding Y. Zhao Y. Ye S. Zhao X. Zhang Y. (2018). Sequentially responsive therapeutic peptide assembling nanoparticles for dual-targeted cancer immunotherapy. Nano Lett. 18, 3250–3258. 10.1021/acs.nanolett.8b01071 29683683

[B15] Choi Y. S. Kwon K. Yoon K. Huh K. M. Kang H. C. (2017). Photosensitizer-mediated mitochondria-targeting nanosized drug carriers: subcellular targeting, therapeutic, and imaging potentials. Int. J. Pharm. 520 (520), 195–206. 10.1016/j.ijpharm.2017.02.013 28179191

[B16] Chu S. Stochaj U. (2020). Exploring near-infrared absorbing nanocarriers to overcome cancer drug resistance. Cancer Drug Resist 3, 302–333. 10.20517/cdr.2020.20 35582453 PMC8992494

[B17] Coussens L. M. Werb Z. (2002). Inflammation and cancer. Nature 26 (420), 860–867. 10.1038/nature01322 PMC2803035 12490959

[B18] Dai T. Ye F. Hu P. Pan X. Chen J. Huang Y. (2020). A strategy for enhanced tumor targeting of photodynamic therapy based on Escherichia Coli-driven drug delivery system. Sci. China Mater 64, 232–240. 10.1007/s40843-020-1363-2

[B19] Daly A. C. Riley L. Segura T. Burdick J. A. (2020). Hydrogel microparticles for biomedical applications. Nat. Rev. Mater 5, 20–43. 10.1038/s41578-019-0148-6 34123409 PMC8191408

[B20] Ethirajan M. Chen Y. Joshi P. Pandey R. K. (2011). The role of porphyrin chemistry in tumor imaging and photodynamic therapy. Chem. Soc. Rev. Jan. 40, 340–362. 10.1039/b915149b 20694259

[B21] Feng Y. K. Tang Q. L. Wang B. Yang Q. Zhang Y. M. Lei L. J. (2024). Targeting the tumor microenvironment with biomaterials for enhanced immunotherapeutic efficacy. J. Nanobiotechnol 22, 737. 10.1186/s12951-024-03005-2 PMC11603847 39605063

[B22] Fu Z. Ni D. Q. Cai S. F. Li H. L. Xiong Y. L. Yang R. (2022). Versatile BP/Pd-FPEI-CpG nanocomposite for “three-in-one” multimodal tumor therapy. Nano Today 46, 101590. 10.1016/j.nantod.2022.101590

[B23] Gao Q. Zhang J. Gao J. Zhang Z. Zhu H. Wang D. (2021). Gold nanoparticles in cancer theranostics. Front. Bioeng. Biotechnol. 9, 647905. 10.3389/fbioe.2021.647905 33928072 PMC8076689

[B24] Ghoochani S. H. Hosseini H. A. Sabouri Z. Soheilifar M. H. Neghab H. K. Hashemzadeh A. (2023). Zn(II) porphyrin-encapsulated MIL-101 for photodynamic therapy of breast cancer cells. Laser Med. Sci. 28, 151. 10.1007/s10103-023-03813-2 37378703

[B25] Glassman P. M. Villa C. H. Ukidve A. Zhao Z. Smith P. Mitragotri S. (2020). Vascular drug delivery using carrier red blood cells: focus on RBC surface loading and pharmacokinetics. pharmaceutics 12, 440. 10.3390/pharmaceutics12050440 32397513 PMC7284780

[B26] Guo Y. Wang M. Zhang Y. Z. Zhao Z. Y. Li J. N. (2025). Advanced hydrogel material for colorectal cancer treatment. Drug Deliv. 32. 10.1080/10717544.2024.2446552 PMC11703513 41410283

[B27] Gupta J. Sharma G. (2024). Nanogel: a versatile drug delivery system for the treatment of various diseases and their future perspective. Drug Deliv. Transl. Res. 15, 455–482. 10.1007/s13346-024-01684-w 39103593

[B28] Han H. S. Choi K. Y. (2021). Advances in nanomaterial-mediated photothermal cancer therapies: toward clinical applications. Biomedicines 16 (9), 305. 10.3390/biomedicines9030305 PMC8002224 33809691

[B29] Han H. S. Choi K. Y. Lee H. Lee M. An J. Y. Shin S. (2016). Gold-nanoclustered hyaluronan nano-assemblies for photothermally maneuvered photodynamic tumor ablation. ACS Nano. Dec 27 (10), 10858–10868. 10.1021/acsnano.6b05113 28024382

[B30] He J. C. Zhang H. J. Zhu J. Zhang X. X. Liu X. X. Ramachandraiah K. (2022). Layer-by-layer synthesis of Au nanorods@metal-organic framework core-shell nanohybrids for magnetic resonance imaging guided photothermal therapy. Mater Today Commun. 33, 104560. 10.1016/j.mtcomm.2022.104560

[B31] Hemmati-Dinarvand M. Ahmadvand H. Seghatoleslam A. (2022). Nitazoxanide and cancer drug resistance: targeting wnt/β-catenin signaling pathway. Arch. Med. Res. 53, 263–270. 10.1016/j.arcmed.2021.12.001 34937659

[B32] Jia X. N. Wang E. R. Wang J. (2025). Rational design of nanozymes for engineered cascade catalytic cancer therapy. Chem. Rev. Jan. 27. 10.1021/acs.chemrev.4c00882 39869790

[B33] Karimi Alavijeh R. Akhbari K. (2024). Cancer therapy by nano MIL-n series of metal-organic frameworks. 0010-8545.503. 10.1016/j.ccr.2023.215643

[B34] Khan A. A. Allemailem K. S. Almatroodi S. A. Almatroudi A. Rahmani A. H. (2020). Recent strategies towards the surface modification of liposomes: an innovative approach for different clinical applications. 3 Biotech. 10, 163. 10.1007/s13205-020-2144-3 PMC7062946 32206497

[B35] Kodiha M. Wang Y. M. Hutter E. Maysinger D. Stochaj U. (2015). Off to the organelles - killing cancer cells with targeted gold nanoparticles. Theranostics 5, 357–370. 10.7150/thno.10657 25699096 PMC4329500

[B36] Li F. Du Y. Liu J. Sun H. Wang J. Li R. (2018). Responsive assembly of upconversion nanoparticles for ph-activated and near-infrared-triggered photodynamic therapy of deep tumors. Adv. Mater. Aug 30, e1802808. 10.1002/adma.201802808 29999559

[B37] Li L. H. Jiang R. T. Yu J. F. Li M. (2024). A Near-Infrared II photo-triggered multifunctional plasmonic hyperthermia immunomodulator for SERS-guided combination cancer immunotherapy. Small. Nov. 20, e2409154. 10.1002/smll.202409154 39564687

[B38] Li W. Zhu X. Zhou X. Wang X. Zhai W. Li B. (2021). An orally available PD-1/PD-L1 blocking peptide OPBP-1-loaded trimethyl chitosan hydrogel for cancer immunotherapy. J. Control. Release 334, 376–388. 10.1016/j.jconrel.2021.04.036 33940058

[B39] Li X. Fan H. Guo T. Bai H. Kwon N. Kim K. H. (2019). Sequential protein-responsive nanophotosensitizer complex for enhancing tumor-specific therapy. ACS Nano 13, 6702–6710. 10.1021/acsnano.9b01100 31184131

[B40] Liang Z. Hao C. Chen C. Ma W. Sun M. Xu L. (2022). Ratiometric FRET encoded hierarchical ZrMOF@Au cluster for ultrasensitive quantifying microRNA *in vivo* . Adv. Mater. Jan. 34, e2107449. 10.1002/adma.202107449 34647652

[B41] Lin R. K. Zhang L. Ye B. W. Wang Y. A. Li Y. D. Jason H. (2023). A multi-functional nano-system combining PI3K-110α/β inhibitor overcomes P-glycoprotein mediated MDR and improves anti-cancer efficiency. Cancer Lett. 563, 216181. 10.1016/j.canlet.2023.216181 37086953

[B42] Liu Y. Zhu X. Wei Z. Feng W. Li L. Ma L. (2021). Customized photothermal therapy of subcutaneous orthotopic cancer by multichannel luminescent nanocomposites. Adv. Mater. Jul 33, e2008615. 10.1002/adma.202008615 34121241

[B43] Llop J. Lammers T. (2021). Nanoparticles for cancer diagnosis, radionuclide therapy and theranostics. ACS Nano 15, 16974–16981. 10.1021/acsnano.1c09139 34748314 PMC7612708

[B44] Mastropietro D. J. Omidian H. Park K. (2012). Drug delivery applications for superporous hydrogels. Expert Opin. Drug Deliv. 9, 71–89. 10.1517/17425247.2012.641950 22145909

[B45] Meng X. Li W. Sun Z. Zhang J. Zhou L. Deng G. (2017). Tumor-targeted small molecule for dual-modal imaging-guided phototherapy upon near-infrared excitation. J. Mater Chem. B 5 (5), 9405–9411. 10.1039/c7tb02496g 32264543

[B46] Mo D. C. Huang J. F. Lin P. Huang S. X. Wang H. L. Luo P. H. (2024). The role of PD-L1 in patients with non-small cell lung cancer receiving neoadjuvant immune checkpoint inhibitor plus chemotherapy: a meta-analysis. Sci. Rep-Uk 14, 26200. 10.1038/s41598-024-78159-y PMC11527982 39482343

[B47] Nakamura T. Kawakami K. Nomura M. Sato Y. Hyodo M. Hatakeyama H. (2022). Combined nano cancer immunotherapy based on immune status in a tumor microenvironment. J. Control. Release 345, 200–213. 10.1016/j.jconrel.2022.03.026 35307507

[B48] Nejabat M. Samie A. Khojastehnezhad A. Hadizadeh F. Ramezani M. Alibolandi M. (2024). Stimuli-responsive covalent organic frameworks for cancer therapy. ACS Appl. Mater Interfaces 16, 51837–51859. 10.1021/acsami.4c07040 39163539

[B49] Nigam M. Mishra A. P. Deb V. K. Dimri D. B. Tiwari V. Bungau S. G. (2023). Evaluation of the association of chronic inflammation and cancer: insights and implications. Biomed. and Pharmacother. 164, 115015. 10.1016/j.biopha.2023.115015 37321055

[B50] Otvagin V. F. Kuzmina N. S. Kudriashova E. S. Nyuchev A. V. Gavryushin A. E. Fedorov A. Y. (2022). Conjugates of porphyrinoid-based photosensitizers with cytotoxic drugs: current progress and future directions toward selective photodynamic therapy. J. Med. Chem. 65, 1695–1734. 10.1021/acs.jmedchem.1c01953 35050607

[B51] Poon W. Zhang Y. N. Ouyang B. Kingston B. R. Wu J. L. Y. Wilhelm S. (2019). Elimination pathways of nanoparticles. ACS Nano 13, 5785–5798. 10.1021/acsnano.9b01383 30990673

[B52] Pustimbara A. Li C. H. Ogura S. (2024). Hemin enhances the 5-aminolevulinic acid-photodynamic therapy effect through the changes of cellular iron homeostasis. Photodiagn Photodyn. Aug 48, 104253. 10.1016/j.pdpdt.2024.104253 38901716

[B53] Racz B. Kristof E. Kincses A. Dominguez-Alvarez E. Spengler G. (2023). Antitumor activity of symmetrical selenoesters in doxorubicin resistant breast cancer. Anticancer Res. 43, 4865–4872. 10.21873/anticanres.16683 37909996

[B54] Ren W. X. Han J. Uhm S. Jang Y. J. Kang C. Kim J. H. (2015). Recent development of biotin conjugation in biological imaging, sensing, and target delivery. ChemComm 51, 10403–10418. 10.1039/c5cc03075g 26021457

[B55] Rommasi F. Esfandiari N. (2021). Liposomal nanomedicine: applications for drug delivery in cancer therapy. Nanoscale Res. Lett. 16, 95. 10.1186/s11671-021-03553-8 34032937 PMC8149564

[B56] Ryman-Rasmussen J. P. Riviere J. E. Monteiro-Riviere N. A. (2006). Penetration of intact skin by quantum dots with diverse physicochemical properties. Toxicol. Sci. 91, 159–165. 10.1093/toxsci/kfj122 16443688

[B57] Sharma N. Bietar K. Stochaj U. (2022). Targeting nanoparticles to malignant tumors. BBA-REV CANCER. Feb 26, 188703. 10.1016/j.bbcan.2022.188703 35227830

[B58] Shukla S. Dalai P. Agrawal-Rajput R. (2024). Metabolic crosstalk: extracellular ATP and the tumor microenvironment in cancer progression and therapy. Cell. Signal. 121, 111281. 10.1016/j.cellsig.2024.111281 38945420

[B59] Singh N. Son S. An J. Kim I. Choi M. Kong N. (2021). Nanoscale porous organic polymers for drug delivery and advanced cancer theranostics. Chem. Soc. Rev. 29 (50), 12883–12896. 10.1039/d1cs00559f 34608468

[B60] Subhan M. A. Yalamarty S. S. K. Filipczak N. Parveen F. Torchilin V. P. (2021). Recent advances in tumor targeting via epr effect for cancer treatment. J. Pers. Med. 11, 571. 10.3390/jpm11060571 34207137 PMC8234032

[B61] Teleanu D. M. Chircov C. Grumezescu A. M. Volceanov A. Teleanu R. I. (2018). Impact of nanoparticles on brain health: an up to Date Overview. J. Clin. Med. 7, 490. 10.3390/jcm7120490 30486404 PMC6306759

[B62] Vaidya F. U. Sufiyan Chhipa A. Mishra V. Gupta V. K. Rawat S. G. Kumar A. (2020). Molecular and cellular paradigms of multidrug resistance in cancer. Cancer Rep. 5, e1291. 10.1002/cnr2.1291 PMC9780431 33052041

[B63] Valdiglesias V. Fernandez-Bertolez N. Kilic G. Costa C. Costa S. Fraga S. (2016). Are iron oxide nanoparticles safe? Current knowledge and future perspectives. J. Trace Elem. Med. Biol. 38, 53–63. 10.1016/j.jtemb.2016.03.017 27056797

[B64] Wang A. Walden M. Ettlinger R. Kiessling F. Gassensmith J. J. Lammers T. (2023). Biomed. metal–organic Framew. Mater. Perspect. challenges 34, 1616–301X. 10.1002/adfm.202308589 PMC7617264 39726715

[B65] Wang D. N. Zhang T. Y. Hu Y. X. Luo Y. N. Li Y. T. Duan D. D. (2024a). Peptide-modified cyclodextrin-based nanosystem for co-delivery of celastrol and siPD-L1 to tumors. Acs Appl. Nano Mater 7, 11432–11444. 10.1021/acsanm.4c01064

[B66] Wang L. P. Ji P. H. Yu J. D. Qiu S. W. An B. L. Huo M. F. (2024b). Hybridized and engineered microbe for catalytic generation of peroxynitrite and cancer immunotherapy under sonopiezo initiation. Sci. Adv. 30, eadp7540. 10.1126/sciadv.adp7540 PMC11524182 39475601

[B67] Wang W. D. Chen C. Ying Y. Lv S. R. Wang Y. Zhang X. (2022). Smart PdH@MnO Yolk-Shell nanostructures for spatiotemporally synchronous targeted hydrogen delivery and oxygen-elevated phototherapy of melanoma. Acs Nano. Mar. 22 (16), 5597–5614. 10.1021/acsnano.1c10450 35315637

[B68] Wang X. Luo J. He L. Cheng X. Yan G. Wang J. (2018). Hybrid pH-sensitive nanogels surface-functionalized with collagenase for enhanced tumor penetration. J. Colloid Interface Sci. 525, 269–281. 10.1016/j.jcis.2018.04.084 29709781

[B69] Wolf K. J. Chen J. Coombes J. D. Aghi M. K. Kumar S. (2019). Dissecting and rebuilding the glioblastoma microenvironment with engineered materials. Nat. Rev. Mater 4, 651–668. 10.1038/s41578-019-0135-y 32647587 PMC7347297

[B70] Wong R. C. H. Lo P. Ng D. K. P. (2019). Stimuli responsive phthalocyanine-based fluorescent probes and photosensitizers. Coord. Chem. Rev. 379, 30–46. 10.1016/j.ccr.2017.10.006

[B71] Wu H. Zhong D. Zhang Z. J. Li Y. C. Zhang X. Li Y. K. (2020). Bioinspired artificial Tobacco Mosaic virus with combined oncolytic properties to completely destroy multidrug-resistant cancer. Adv. Mater. Mar. 32, e1904958. 10.1002/adma.201904958 31961987

[B72] Wu X. Q. Yan J. Han X. Q. Zheng R. X. Song P. P. Wang Y. J. (2022). Core-shell nanomaterials engineered to reverse cancer multidrug resistance by immunotherapy and promote photo-responsive chemotherapy. Chem. Eng. J. 1, 132329. 10.1016/j.cej.2021.132329

[B73] Xiao Q. Zheng X. Bu W. Ge W. Zhang S. Chen F. (2013). A core/satellite multifunctional nanotheranostic for *in vivo* imaging and tumor eradication by radiation/photothermal synergistic therapy. J. Am. Chem. Soc. Sep. 4 (135), 13041–13048. 10.1021/ja404985w 23924214

[B74] Xiong F. Nirupama S. Sirsi S. R. Lacko A. Hoyt K. (2017). Ultrasound-stimulated drug delivery using therapeutic reconstituted high-density lipoprotein nanoparticles. Nanotheranostics 1, 440–449. 10.7150/ntno.21905 29188177 PMC5704009

[B75] Xu F. Huang X. Wang Y. Zhou S. (2020). A size-changeable collagenase-modified nanoscavenger for increasing penetration and retention of nanomedicine in deep tumor tissue. Adv. Mater. Apr 32, e1906745. 10.1002/adma.201906745 32105374

[B76] Yang E. L. Wang W. Y. Liu Y. Q. Yi H. Lei A. W. Sun Z. J. (2024). Tumor-targeted catalytic immunotherapy. Adv. Mater. Dec 15, e2413210. 10.1002/adma.202413210 39676382

[B77] Yang N. Xiao W. Y. Song X. J. Wang W. J. Dong X. C. (2020). Recent advances in tumor microenvironment hydrogen peroxide-responsive materials for cancer photodynamic therapy. Nano-Micro Lett. Jan. 12, 15. 10.1007/s40820-019-0347-0 PMC7770924 34138092

[B78] Yu Y. Yang X. Reghu S. Kaul S. C. Wadhwa R. Miyako E. (2020). Photothermogenetic inhibition of cancer stemness by near-infrared-light-activatable nanocomplexes. Nat. Commun. 17, 4117. 10.1038/s41467-020-17768-3 PMC7431860 32807785

[B79] Yuan H. Z. Yang H. Yuan P. Wang T. T. Zhou Q. (2023). Carbon quantum dots as drug carriers for tumor-associated macrophage repolarization following photothermal therapy. Scienceasia. Aug 49, 627–634. 10.2306/scienceasia1513-1874.2023.060

[B80] Yuan X. W. Li K. S. Lv F. F. Li N. Zhang L. R. Zhao S. L. (2024). Inhibiting effect of cationic procyanidin nanoparticles on drug-resistant oral squamous cell carcinoma cell lines. Lett. Drug Des. Discov. 21, 782–789. 10.2174/1570180820666230206125313

[B81] Yurdabak Karaca G. Kuralay F. Bingol Ozakpinar O. Uygun E. Koc U. Ulusoy S. (2021). RETRACTED ARTICLE: catalytic Au/PEDOT/Pt micromotors for cancer biomarker detection and potential breast cancer treatment. Appl. Nanosci. 13, 367. 10.1007/s13204-021-01735-5

[B82] Zhang H. (2016). Erythrocytes in nanomedicine: an optimal blend of natural and synthetic materials. Biomater. Sci. 21 (4), 1024–1031. 10.1039/c6bm00072j 27090487

[B83] Zhang L. Li Y. D. Wang Q. C. Chen Z. Li X. Y. Wu Z. X. (2020). The PI3K subunits, P110α and P110β are potential targets for overcoming P-gp and BCRP-mediated MDR in cancer. Mol. Cancer 19, 10. 10.1186/s12943-019-1112-1 31952518 PMC6966863

[B84] Zhang R. Zhang C. Chen C. Tian M. Chau J. H. C. Li Z. (2023). Autophagy-activated self-reporting photosensitizer promoting cell mortality in cancer starvation therapy. Adv. Sci. (Weinh) 10 (Jun), e2301295. 10.1002/advs.202301295 37083241 PMC10288242

[B85] Zhang X. Ameer F. S. Azhar G. Wei J. Y. (2021). Alternative splicing increases sirtuin gene family diversity and modulates their subcellular localization and function. Int. J. Mol. Sci. 6, 473. 10.3390/ijms22020473 PMC7824890 33418837

[B86] Zhao M. Zhuang H. Li B. Chen M. Chen X. (2023). *In situ* transformable nanoplatforms with supramolecular cross-linking triggered complementary function for enhanced cancer Photodynamic Therapy. Adv. Mater. May 35, e2209944. 10.1002/adma.202209944 36856448

[B87] Zhou Q. Li Z. J. Xi Y. M. (2024). EV-mediated intercellular communication in acute myeloid leukemia: transport of genetic materials in the bone marrow microenvironment. Exp. Hematol. May 133, 104175. 10.1016/j.exphem.2024.104175 38311165

[B88] Zhu M. S. Liu Q. Q. Chen Z. B. Liu J. M. Zhang Z. X. Tian J. W. (2025). Rational design of dual-targeted nanomedicines for enhanced vascular permeability in low-permeability tumors. Acs Nano 19 (19), 3424–3438. 10.1021/acsnano.4c12808 39797815

